# Development and validation of a nomogram with an autophagy-related gene signature for predicting survival in patients with glioblastoma

**DOI:** 10.18632/aging.102566

**Published:** 2019-12-17

**Authors:** Zihao Wang, Lu Gao, Xiaopeng Guo, Chenzhe Feng, Wei Lian, Kan Deng, Bing Xing

**Affiliations:** 1 Department of Neurosurgery, Peking Union Medical College Hospital, Chinese Academy of Medical Sciences and Peking Union Medical College, Dongcheng, Beijing 100730, P.R. China; 2 China Pituitary Disease Registry Center, Chinese Pituitary Adenoma Cooperative Group, Dongcheng, Beijing 100730, P.R. China

**Keywords:** glioblastoma, GSEA, autophagy, gene signature, prognostic model

## Abstract

Glioblastoma (GBM) is the most common brain tumor with significant morbidity and mortality. Autophagy plays a vital role in GBM development and progression. We aimed to establish an autophagy-related multigene expression signature for individualized prognosis prediction in patients with GBM. Differentially expressed autophagy-related genes (DE-ATGs) in GBM and normal samples were screened using TCGA. Univariate and multivariate Cox regression analyses were performed on DE-ATGs to identify the optimal prognosis-related genes. Consequently, NRG1 (HR=1.142, P=0.008), ITGA3 (HR=1.149, P=0.043), and MAP1LC3A (HR=1.308, P=0.014) were selected to establish the prognostic risk score model and validated in the CGGA validation cohort. GSEA revealed that these genes were mainly enriched in cancer- and autophagy-related KEGG pathways. Kaplan-Meier survival analysis demonstrated that patients with high risk scores had significantly poorer overall survival (OS, log-rank P= 6.955×10^-5^). The autophagy signature was identified as an independent prognostic factor. Finally, a prognostic nomogram including the autophagy signature, age, pharmacotherapy, radiotherapy, and IDH mutation status was constructed, and TCGA/CGGA-based calibration plots indicated its excellent predictive performance. The autophagy-related three-gene risk score model could be a prognostic biomarker and suggest therapeutic targets for GBM. The prognostic nomogram could assist individualized survival prediction and improve treatment strategies.

## INTRODUCTION

Glioma is the most common primary tumor of the central nervous system, with an average annual age-adjusted incidence rate of 6.0 per 100,000 in the United States from 2010 to 2014 [1]. According to the 2016 World Health Organization (WHO) classification, glioblastoma (GBM), corresponding to grade IV glioma, is the most commonly occurring type of glioma [2]. Despite considerable advances in the development of treatments for GBM, including surgery, radiotherapy, chemotherapy, targeted therapy, and immunotherapy, the optimal management strategy remains controversial [3]. Notably, GBM patients generally exhibit significant morbidity and mortality, with a 5-year overall survival (OS) rate of approximately 5% [1]. Clinicopathologic parameters, including age and resection extent, and various molecular alterations have been reported as the prognostic factors for GBM in the literature [4–6]. Although numerous clinical and molecular studies in GBM have been reported in recent years, the prognostic biomarkers and predictors of therapeutic responses for GBM are still not clearly elucidated.

Autophagy is a lysosomal degradation pathway that is essential for survival, differentiation, development, and homeostasis [7] and has been reported to play a key role in diverse pathologies, especially cancer [8]. By eliminating damaged proteins and organelles, autophagy can suppress early-stage development of cancer, thereby mitigating cellular damage and limiting chromosomal instability [9, 10]. However, autophagy can also promote tumor growth by providing nutritional elements under low oxygen and low nutrient conditions [11]. In most cases, autophagy is thought to suppress early tumorigenesis and promote the development of existing tumors [12]. Previous studies have investigated the roles of certain autophagy-related genes (ATGs) in the development and progression of glioma. These ATGs may be regulated by—and, in turn, regulate—multiple signaling pathways, many of which are dysregulated in GBM and targetable with various inhibitors [12, 13]. Therefore, ATGs are promising therapeutic targets and prognostic predictors in GBM.

With the rapid development of large-scale genome-sequencing technologies, numerous studies have investigated large numbers of molecular biomarkers for GBM, including 1p/19q codeletion, telomerase reverse transcriptase (TERT) promoter mutations, tumor protein 53 (TP53) mutations, X-linked helicase II (ATRX) mutations, and isocitrate dehydrogenase (IDH) mutation [14–16]. Emerging evidence demonstrates that certain single genes cannot completely represent the characteristics of tumors, whereas global gene expression patterns of multiple genes can serve as excellent molecular biomarkers that allow early diagnosis, subgroup classification, risk stratification, prognosis prediction, and therapeutic targeting in GBM [17–19]. However, global expression patterns based on ATGs have not previously been recognized in GBM.

In this study, by assessing the global gene expression profile, we aimed to investigate and validate an autophagy-related multiple gene expression signature that can predict prognosis and suggest therapeutic targets in GBM. Moreover, we combined both the autophagy signature and clinical parameters to establish a novel promising prognostic nomogram model with more accurate predictive ability than clinical risk factors for GBM patients.

## RESULTS

### Identification of DE-ATGs and enrichment analysis

**Figure 1:**
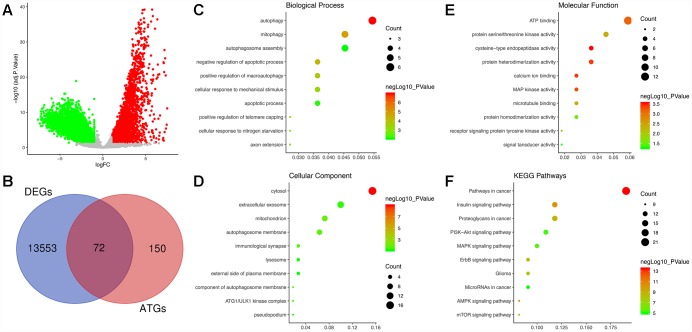
**Identification of differentially expressed autophagy-related genes (DE-ATGs) in glioblastoma (GBM) and enrichment analysis.** (**A**) Volcano plot of DEGs in tumor and normal samples of The Cancer Genome Atlas (TCGA) dataset. The vertical axis indicates the -log (adjusted P value [adj. P value]), and the horizontal axis indicates the log2 (fold change [FC]). The red dots represent upregulated genes, and the green dots represent downregulated genes (adj. P value <0.01 and |log2(FC)|>1). (**B**) Venn diagram showing the 72 DE-ATGs (the intersection of the DEGs and ATGs). (**C**) Biological processes enriched in the DE-ATGs. (**D**) Cellular components enriched in the DE-ATGs. (**E**) Molecular functions enriched in the DE-ATGs. (**F**) Kyoto Encyclopedia of Genes and Genomes (KEGG) pathways enriched in the DE-ATGs.

Following analysis of the TCGA GBM dataset using edgeR, a total of 13625 DEGs were identified in GBM and normal samples, and these genes are displayed in the volcano plot (Figure 1A). As shown by the Venn diagrams in Figure 1B, the seventy-two significant DE-ATGs (the intersection of the DEGs and ATGs) were selected for further analysis.

GO analysis, including the biological process (BP), cellular component (CC) and molecular function (MF) categories, was performed on the DE-ATGs. In the BP category, the DE-ATGs were significantly enriched in the terms autophagy, mitophagy and autophagosome assembly (Figure 1C). In the CC category, the DE-ATGs were significantly enriched in the terms cytosol, extracellular exosome, mitochondrion and autophagosome membrane (Figure 1D). In the MF category, the DE-ATGs were significantly enriched in the terms ATP binding, protein serine/threonine kinase activity, and cysteine-type endopeptidase activity (Figure 1E). In addition, KEGG pathway analysis revealed that the DE-ATGs were mainly enriched in pathways in cancer, insulin signaling pathway and proteoglycans in cancer.

### Identification of prognosis-related ATGs

**Figure 2:**
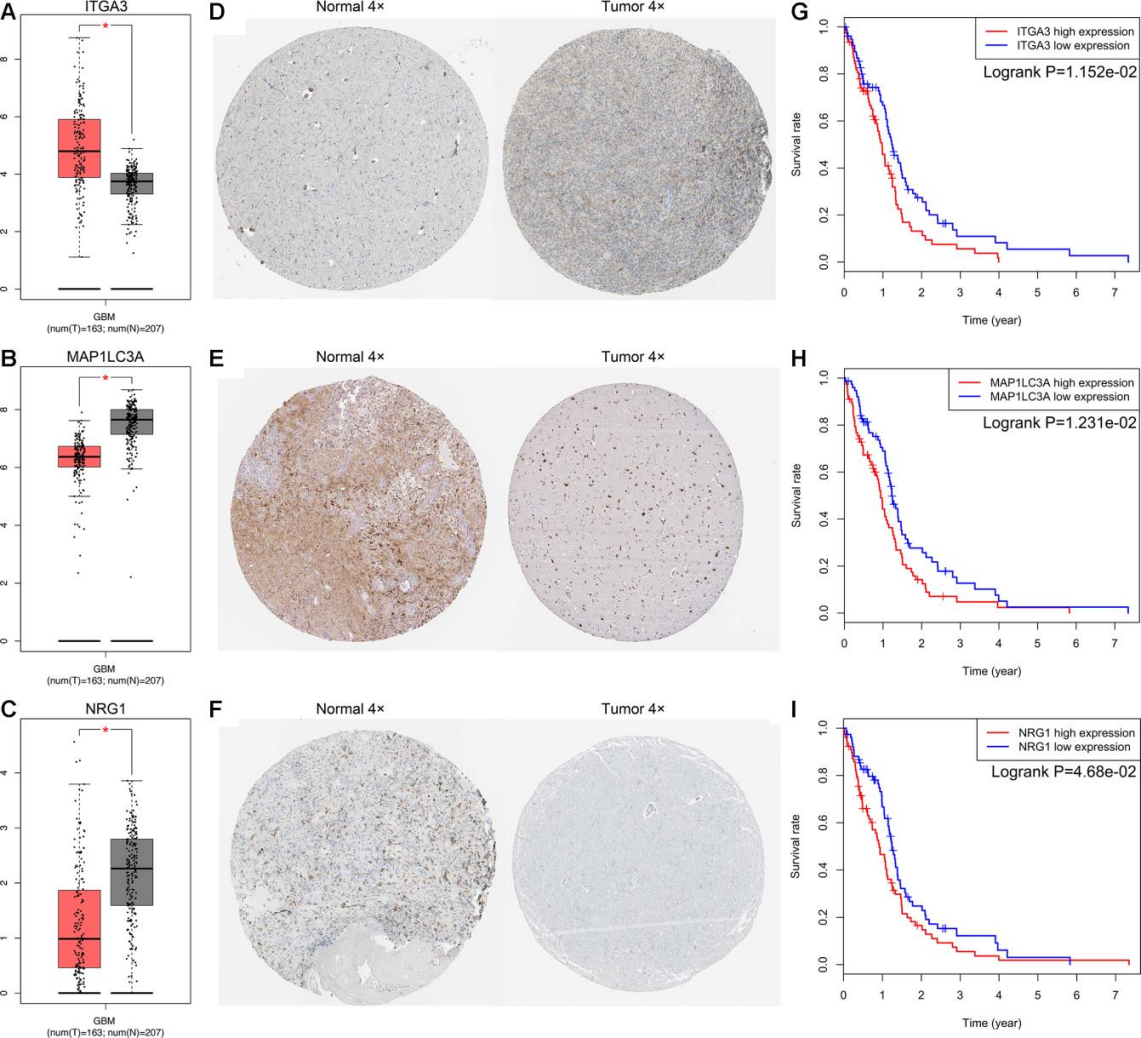
**Expression and survival analysis for ITGA3, MAP1LC3A and NRG1 in GBM.** The expression levels of ITGA3 (**A**), MAP1LC3A (**B**) and NRG1 (**C**) in tumor and normal tissues were validated in the GEPIA database, which included 163 GBM samples and 207 normal samples. The red box on the left was tumor group, and the gray box on the right was normal group. The expression profiles of the proteins encoded by ITGA3 (**D**), MAP1LC3A (**E**) and NRG1 (**F**) in normal and tumor tissues using clinical specimens from the Human Protein Profiles. K-M OS curves based on the expression levels of ITGA3 (**G**), MAP1LC3A (**H**) and NRG1 (**I**) in patients with GBM in the TCGA dataset.

By performing univariate Cox regression analysis on the 72 candidate genes in the TCGA cohort consisting of 155 GBM patients, we identified 9 prognosis-related genes, which were indicated to have significant prognostic value (P<0.05). Subsequent multivariate Cox regression analysis indicated that only 3 genes—Neuregulin 1 (NRG1, HR 1.142, P=0.008), Integrin Subunit Alpha 3 (ITGA3, HR 1.149, P=0.043), and Microtubule-Associated Protein 1 Light Chain 3 Alpha (MAP1LC3A, HR 1.308, P=0.014)—exhibited significant prognostic value for GBM (Supplementary Table 1). The differential expression of the above three genes in tumor and normal tissues was further validated in the Gene Expression Profiling Interactive Analysis (GEPIA) database, which included 163 GBM samples and 207 normal samples, revealing that ITGA3 was significantly upregulated but NRG1 and MAP1LC3A were significantly downregulated in GBM tissues (Figure 2A–2C) [20]. Then, we analyzed the expression of the proteins encoded by the three genes using clinical specimens from the Human Protein Profiles (https://www.proteinatlas.org) [21]. MAP1LC3A was moderately positive but ITGA3 and NRG1 were weakly positive in GBM tissue relative to their expression levels in normal tissue (Figure 2D–2F). In addition, K-M survival curves were constructed to assess the associations between the expression levels of the prognosis-related genes and OS, and the results indicated that the ITGA3, NRG1 and MAP1LC3A low-expression group (log-rank P = 0.012, 0.012, and 0.047) had a better prognosis (Figure 2G–2I).

### Construction of the ATG-based prognostic risk score model (autophagy signature)

**Figure 3:**
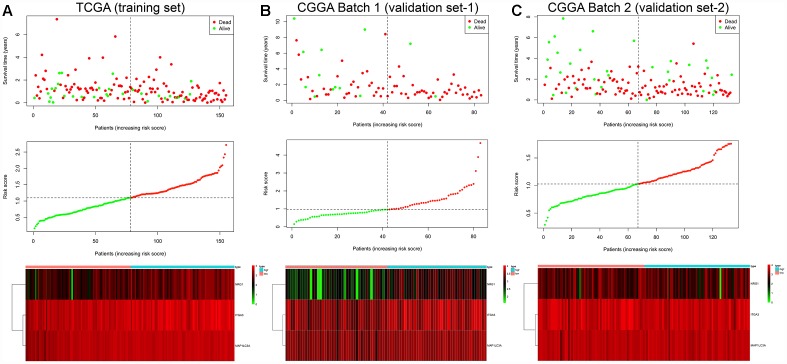
Risk score analysis of the GBM autophagy signature in the TCGA training cohort (**A**), CGGA Batch-1 validation cohort (**B**), and CGGA Batch-2 validation cohort (**C**). Upper panel: patient survival status and time distributed by risk score. Middle panel: risk score curve of the autophagy signature. Bottom panel: heatmap of NRG1, ITGA3, and MAP1LC3A expression in GBM samples. The colors from green to red indicate the expression level from low to high. The dotted line indicates the individual inflection point of the risk score curve, by which the patients were categorized into low-risk and high-risk groups.

**Figure 4:**
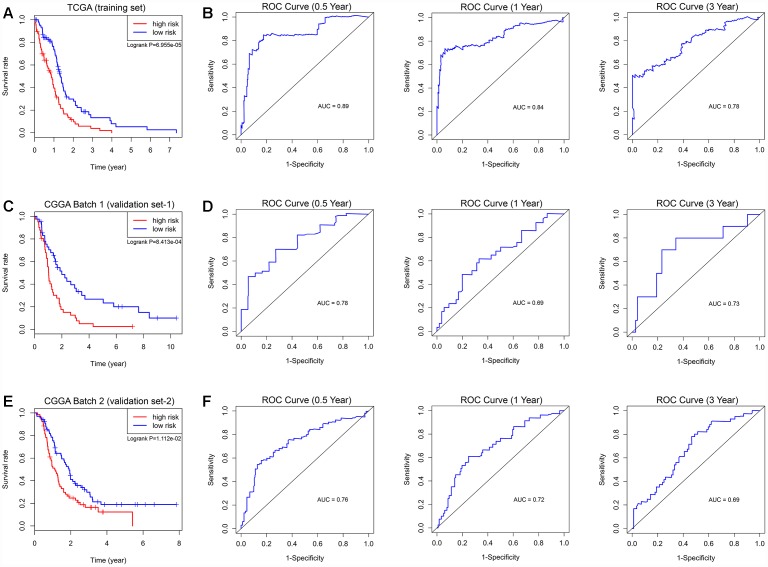
**Survival analysis and prognostic performance of the autophagy-related risk score model in GBM.** K-M survival curve of the risk score for patient OS in the TCGA training cohort (**A**), CGGA Batch-1 validation cohort (**C**), and CGGA Batch-2 validation cohort (**E**). The high-risk groups had significantly poorer OS rates than the low-risk groups. The prognostic performance of the autophagy signature demonstrated by the time-dependent ROC curve for predicting the 0.5-, 1-, and 3-year OS rates in the TCGA training cohort (**B**), CGGA Batch-1 validation cohort (**D**), and CGGA Batch-2 validation cohort (**F**).

The prognostic risk score model was established with the following formula: risk score = expression level of NRG1 × 0.132 + expression level of ITGA3 × 0.139 + expression level of MAP1LC3A × 0.269. Subsequently, we calculated the prognostic risk score for each patient in the TCGA training set. All patients were divided into the high-risk (high risk score) or the low-risk (low risk score) group using the median risk score as the cutoff (Figure 3A). In addition, K-M survival analysis demonstrated that patients with high risk scores had significantly poorer OS than patients with low risk scores (log-rank P = 6.955×10^-5^). The 6-month OS rates of the high-risk and low-risk groups were 64.3% and 84.2%, respectively. The 1-year OS rates of the high-risk and low-risk groups were 39.5% and 73.4%, respectively. The 3-year OS rates of the high-risk and low-risk groups were 3.9% and 13.3%, respectively (Figure 4A). The C-index of the ATG-based prognostic model for OS prediction was 0.782 (95% CI, 0.743 to 0.821; P = 5.13×10^-11^). Furthermore, the autophagy signature showed favorable predictive ability of the 0.5-, 1- and 3-year OS rates, with AUC values of 0.89, 0.84 and 0.78, respectively, in the TCGA training set (Figure 4B). In addition, when patients were stratified by different clinicopathologic parameters, the autophagy signature remained a significant prognostic factor in the TCGA training set and in the CGGA validation sets (Supplementary Figure 1).

### Evaluation of the prognostic autophagy signature in external validation cohorts

To confirm that the prognostic autophagy signature had similar predictive values in different populations, we then used it to predict OS in two independent external validation cohorts using the median risk score as the cutoff. As shown in Figure 3B, a total of 83 patients in the CGGA Batch-1 set (validation set-1) were classified into a low-risk group (n = 42) and a high-risk group (n = 41), and the OS of the GBM patients in the high-risk group was significantly lower than that of the patients in the low-risk group (log-rank P = 8.413×10^-4^; Figure 4C). The autophagy signature constructed with the TCGA training set also showed a favorable predictive ability for the 0.5-, 1- and 3-year OS rates, with AUC values of 0.78, 0.69 and 0.73, respectively, in the CGGA validation set-1 (Figure 4D). In addition, as shown in Figure 3C, a total of 133 patients in the CGGA Batch-2 set (validation set-2) were classified into a low-risk group (n = 67) and a high-risk group (n = 66), and the OS of the GBM patients in the high-risk group was significantly lower than that of patients in the low-risk group (log-rank P = 1.112×10^-2^; Figure 4E). The autophagy signature generated by the TCGA training set also showed a favorable predictive ability for the 0.5-, 1- and 3-year OS rates, with AUC values of 0.76, 0.72 and 0.69, respectively, in the CGGA validation set-2 (Figure 4F).

### Determination of the autophagy signature as an independent prognostic factor

Table 1 shows the demographics and clinicopathologic characteristics of GBM patients in the TCGA training cohort and CGGA validation cohorts based on the autophagy signature. Then, we performed univariate and multivariate Cox regression analyses to evaluate the prognostic significance of the autophagy signature combined with various clinicopathologic parameters (Table 2). In the TCGA training cohort, univariate analysis indicated that the autophagy signature (P = 9.54×10^-5^), age (P = 9.67×10^-3^), new event occurrence (P = 4.59×10^-3^), pharmacotherapy (P = 1.25×10^-4^), radiotherapy (P = 1.55×10^-6^) and IDH status (P = 8.39×10^-3^) were significantly associated with OS. Subsequent multivariate analysis indicated that the autophagy signature (P = 1.24×10^-4^), age (P = 0.028), pharmacotherapy (P = 0.030), radiotherapy (P = 6.36×10^-5^) and IDH status (P = 0.029) were significantly correlated with OS. Therefore, the prognostic autophagy signature constructed by the TCGA training set was an independent prognostic factor for GBM. In addition, following the univariate and multivariate analyses, the autophagy signature was also proven to be an independent prognostic factor in both the CGGA Batch-1 and Batch-2 validation cohorts (Table 2). The K-M survival curves and log-rank test for all these clinicopathological variables in the TCGA training set and CGGA validation sets are shown in Supplementary Figure 2.

**Table 1:** Demographics and clinicopathologic characteristics of GBM patients in the TCGA training cohort and CGGA validation cohort based on the autophagy signature.

**Variables**	**TCGA (Training set)**			**CGGA Batch 1 (Validation set-1)**			**CGGA Batch 2 (Validation set-2)**		
	**Total (n=155)**	**Low risk (n=78)**	**High risk (n=77)**	**Total (n=83)**	**Low risk (n=42)**	**High risk (n=41)**	**Total (n=133)**	**Low risk (n=67)**	**High risk (n=66)**
Age									
<= 50 years	38	24	14	40	22	18	54	28	26
> 50 years	117	54	63	43	20	23	79	39	40
Sex									
Female	54	26	28	32	19	13	54	29	25
Male	101	52	49	51	23	28	79	38	41
New event									
None or NA	66	30	36	NA			NA		
Yes	89	48	41	NA			NA		
KPS									
< 80	33	19	14	NA			NA		
>= 80	83	41	42	NA			NA		
NA	39	18	21	NA			NA		
Pharmaceutical therapy								
CT only	65	34	31	23 (No)	8	15	17 (No)	11	6
CT + TMT	27	13	14	57 (Yes)	32	25	111(Yes)	54	57
CT + HT	21	14	7	3 (NA)	2	1	5 (NA)	2	3
Others	5	4	1	-			-		
No or NA	37	13	24	-			-		
Radiation therapy									
No	23	7	16	10	6	4	17	9	7
Yes	125	66	59	70	34	36	113	56	57
NA	7	5	2	3	2	1	4	2	2
Surgery									
Biopsy only	16	10	6	NA			NA		
Tumor resection	139	68	71	NA			NA		
IDH status									
Wildtype	147	70	77	72	33	39	103	50	53
Mutant	8	8	0	11	9	2	30	17	13
1p/19q status									
Non-codeletion	NA			82	41	41	103	63	40
Codeletion	NA			0	0	0	5	4	1
NA	NA			1	1	0	25	0	25

NA, not available; KPS, Karnofsky performance score; CT, chemotherapy; TMT, targeted molecular therapy; HT, hormone therapy.

“New event” included progression and recurrence. “Others” in pharmaceutical therapy included CT + TMT + HT, CT + TMT + Immunotherapy, and CT + Immunotherapy.

**Table 2:** Univariate and multivariate cox proportional hazards analysis of clinical parameters and risk score of GBM patients in the TCGA training cohort and CGGA validation cohorts.

**Variables**	**TCGA (Training set)**				**CGGA Batch 1 (Validation set-1)**				**CGGA Batch 2 (Validation set-2)**			
	**Univariate Analysis**		**Multivariate analysis**		**Univariate Analysis**		**Multivariate analysis**		**Univariate Analysis**		**Multivariate analysis**	
	**HR (95% CI)**	**P**	**HR (95% CI)**	**P**	**HR (95% CI)**	**P**	**HR (95% CI)**	**P**	**HR (95% CI)**	**P**	**HR (95% CI)**	**P**
Age												
<= 50 years	Reference		Reference		Reference		Reference		Reference			
> 50 years	1.80 (1.15–2.82)	9.67e-3	1.31 (1.03–2.64)	0.028	1.27 (1.09–2.04)	0.032	1.28 (1.07–2.09)	0.033	1.32 (0.88–1.98)	0.18		
Sex												
Female	Reference				Reference				Reference			
Male	0.96 (0.66–1.40)	0.835			1.25 (0.76–2.05)	0.389			0.83 (0.56–1.23)	0.357		
New event		
None or NA	Reference		Reference		NA				NA			
Yes	0.59 (0.40–0.85)	4.59e-3	0.78 (0.51–1.21)	0.272	NA				NA			
KPS												
< 80	Reference				NA				NA			
>= 80	0.77 (0.49–1.23)	0.276			NA				NA			
NA	0.89 (0.53–1.52)	0.684			NA				NA			
Pharmaceutical therapy												
CT only	Reference		Reference		(No) Reference	Reference		(No) Reference	Reference	
CT + TMT	0.92 (0.54–1.55)	0.743	0.94 (0.55–1.62)	0.828	(Yes) 0.39 (0.23–0.67)	6.19e-4	0.41 (0.24–0.70)	1.31e-3	(Yes) 0.51 (0.28–0.92)	0.026	0.81 (0.30–0.96)	0.037
CT + HT	1.43 (0.84–2.44)	0.190	1.41 (0.82-2.43)	0.216	(NA) 0.69 (0.20–2.36)	0.558	0.85 (0.20–3.59)	0.827	(NA) 1.18 (0.33–4.20)	0.794	2.06 (0.22–8.97)	0.523
Others	1.14 (0.45-2.89)	0.784	1.37 (0.75–2.50)	0.260	-				-			
No or NA	2.47 (1.56–3.91)	1.25e-4	1.73 (1.67–4.45)	0.030	-				-			
Radiation therapy												
No	Reference		Reference		Reference		Reference		Reference		Reference	
Yes	0.31 (0.19–0.50)	1.55e-6	0.26 (0.13–0.50)	6.36e-5	0.81 (0.50–0.94)	0.049	0.78 (0.34–0.97)	0.045	0.33 (0.18–0.61)	4.72e-4	0.29 (0.10–0.82)	0.019
NA	0.60 (0.24–1.50)	0.275	0.44 (0.17–1.10)	0.079	0.58 (0.40–6.19)	0.513	0.58 (0.40–6.19)	0.513	0.69 (0.15–3.08)	0.622	0.31 (0.02–4.21)	0.376
Surgery												
Biopsy only	Reference				NA				NA			
Tumor resection	0.95 (0.53–1.69)	0.852			NA				NA			
IDH status												
Wildtype	Reference		Reference		Reference				Reference		Reference	
Mutant	0.26 (0.09–0.71)	8.39e-3	0.28 (0.09–0.88)	0.029	0.64 (0.32–1.30)	0.218			0.43 (0.25–0.74)	2.36e-3	0.40 (0.23–0.68)	8.33e-4
1p/19q status												
Non-codeletion	NA				NA				Reference			
Codeletion	NA				NA				0.77 (0.24–2.43)	0.650		
NA	NA				NA				1.11 (0.66–1.87)	0.704		
Autophagy signature												
Low risk	Reference		Reference		Reference		Reference		Reference		Reference	
High risk	2.06 (1.43–2.96)	9.54e-5	2.12 (1.45–3.12)	1.24e-4	2.27 (1.39–3.72)	1.12e-3	2.28 (1.35–3.86)	0.002	1.66 (1.12–2.46)	0.012	1.75 (1.17–2.61)	0.006

HR, hazard ratio; CI, confidence interval; NA, not available; KPS, Karnofsky performance score; CT, chemotherapy; TMT, targeted molecular therapy; HT, hormone therapy.

“New event” included progression and recurrence. “Others” in pharmaceutical therapy included CT + TMT + HT, CT + TMT + Immunotherapy, and CT + Immunotherapy.

All statistical tests were two-sided.

### GSEA

**Figure 5:**
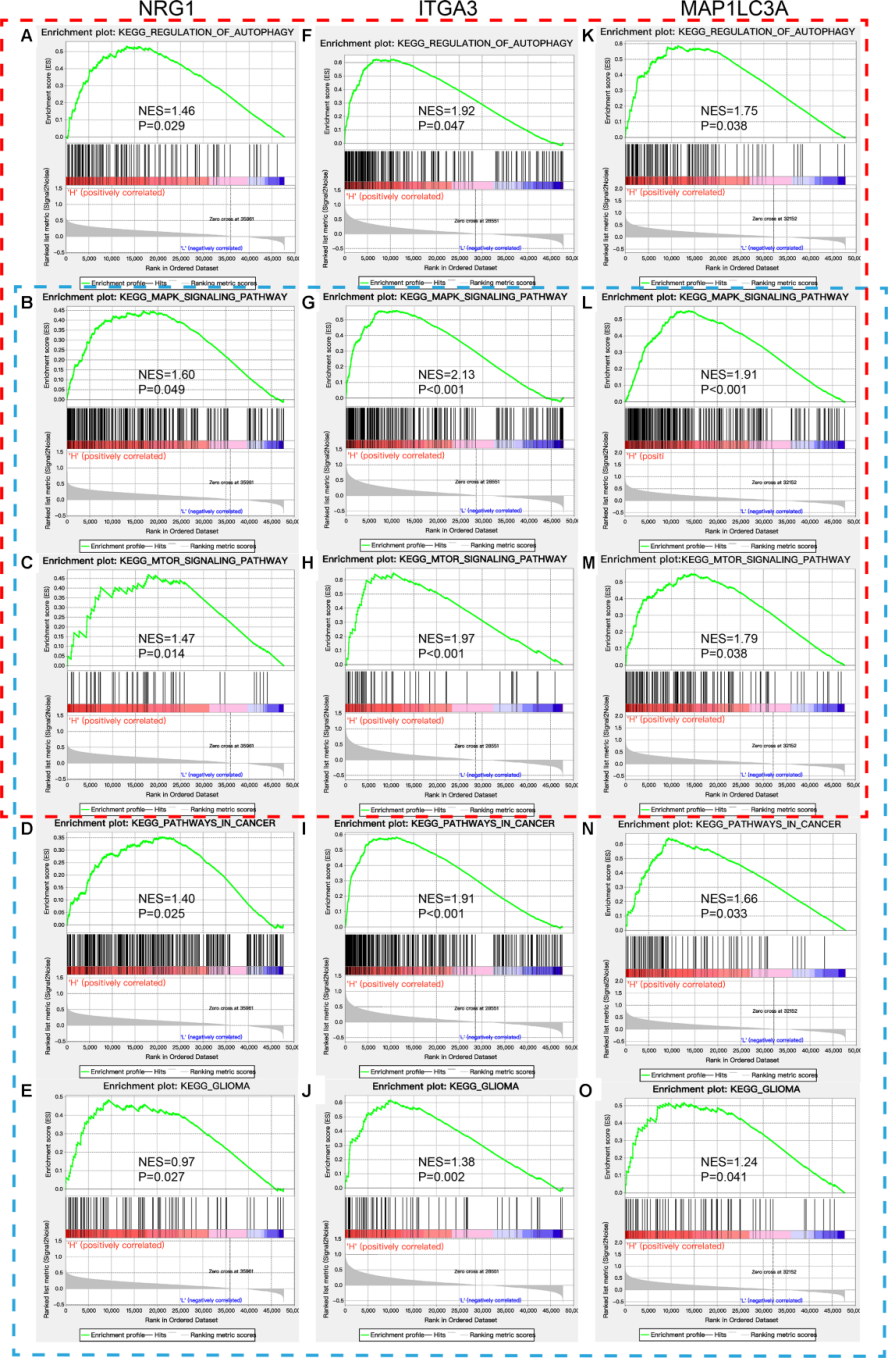
**GSEA of NRG1, ITGA3, and MAP1LC3A in the TCGA GBM cohort.** Red box: regulation of autophagy and autophagy-related KEGG pathways (**A**–**C**, **F**–**H**, and **K**–**M**). Blue box: pathways in cancer and their related KEGG pathways, including glioma (**B**–**E**, **G**–**J**, and **L**–**O**).

GSEA revealed that the DE-ATGs in the high ITGA3, MAP1LC3A, and NRG1 expression groups in the TCGA GBM cohort were mainly enriched in KEGG pathways related to autophagy and cancer. The significantly enriched autophagy-related pathways included regulation of autophagy, MAPK signaling pathway, endocytosis, and insulin signaling pathway. The significantly enriched cancer-related pathways included pathways in cancer, MAPK signaling pathway, mTOR signaling pathway, and glioma (Figure 5, Supplementary Table 2). In addition, GSEA was performed in the ATG-based high-risk and low-risk groups in the TCGA GBM cohort, and the DE-ATGs were also significantly enriched in the pathways related to autophagy and cancer. The DE-ATGs in the high-risk group were enriched mainly in the lysosome, cytokine-cytokine receptor interaction, and focal adhesion pathways. The DE-ATGs in the low-risk group were enriched mainly in MAPK signaling pathway, endocytosis, and insulin signaling pathway (Supplementary Figure 3, Supplementary Table 3). In summary, the defined DE-ATGs contribute to vital cancer and autophagy-related KEGG pathways, which can provide strong evidence for a cancer-targeted treatment for GBM.

### Construction and validation of the nomogram

**Figure 6:**
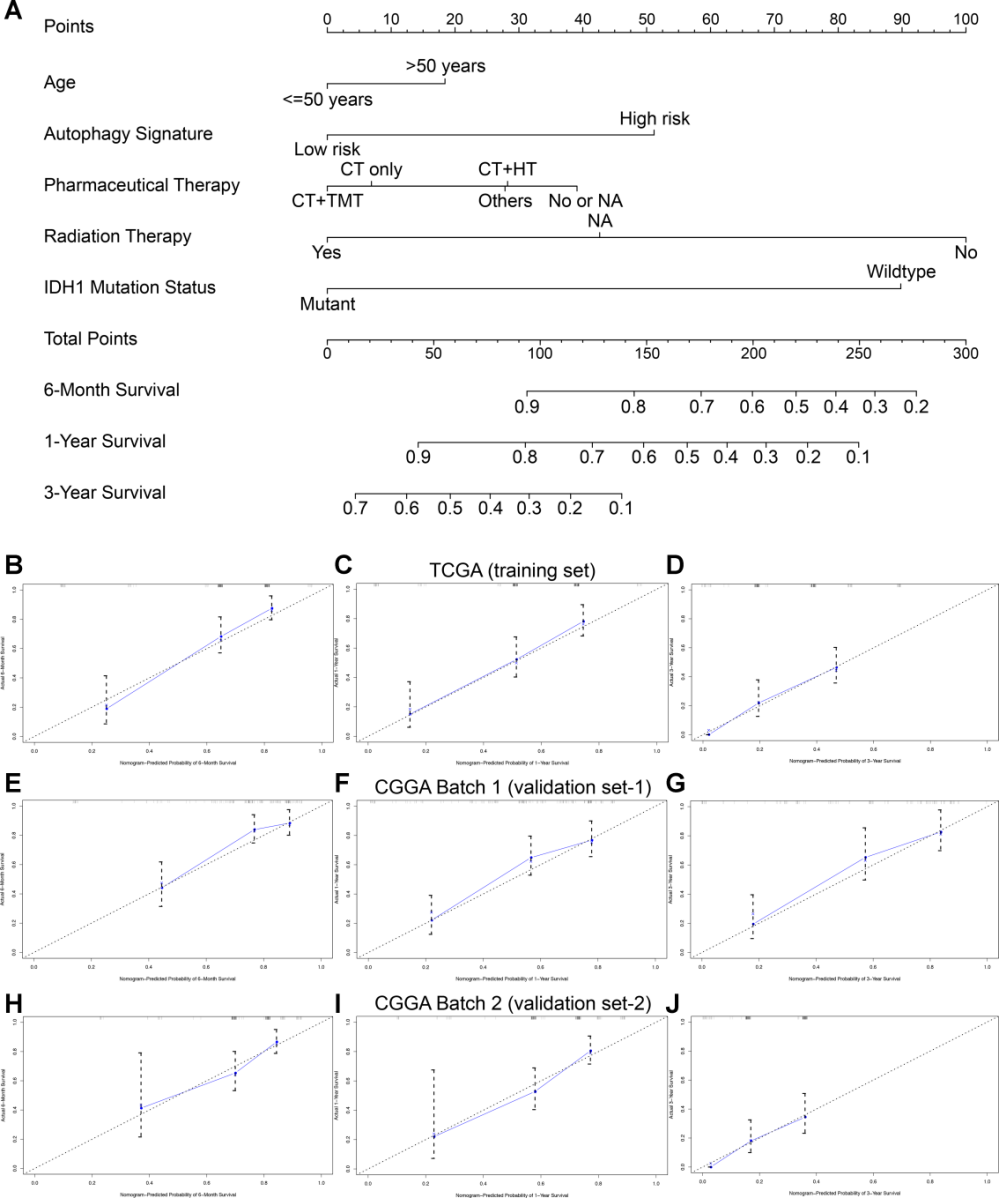
**Nomogram to predict the 0.5-, 1-, and 3-year survival probability of patients with GBM.** (**A**) Prognostic nomogram to predict the survival of GBM patients based on the TCGA training set. Calibration curves of the nomogram for predicting survival at 0.5, 1, and 3 years in the TCGA training cohort (**B**–**D**), CGGA Batch-1 validation cohort (**E**–**G**), and CGGA Batch-2 validation cohort (**H**–**J**). The actual survival is plotted on the y-axis; the nomogram-predicted probability is plotted on the x-axis.

To establish a clinically applicable method for predicting the prognosis of GBM patients, we established a prognostic nomogram to predict the survival probability at 0.5, 1, and 3 years based on the TCGA training set. Five independent prognostic parameters, including age, autophagy signature, pharmacotherapy, radiotherapy and IDH status, were enrolled in the prediction model (Figure 6A). The C-index of the nomogram was 0.832 (95% CI, 0.793 to 0.871; P = 3.013×10^-10^). The calibration plots (Figure 6B–6D) show excellent agreement between the nomogram prediction and actual observation in terms of the 0.5-, 1- and 3-year survival rates in the TCGA cohort. The nomogram also showed a favorable predictive ability for the 0.5-, 1- and 3-year OS rates, with AUC values of 0.807, 0.739 and 0.787, respectively (Supplementary Figure 4). These findings suggest the appreciable reliability of the nomogram. In addition, in the two CGGA external validation cohorts, the C-indexes of the nomogram for predicting OS were 0.737 and 0.721. The calibration plots also demonstrate excellent agreement between prediction and observation for the 0.5-, 1- and 3-year OS probabilities of the patients in CGGA Batch-1 (Figure 6E–6G) and Batch-2 (Figure 6H–6J). The AUC values demonstrated that the nomogram also has favorable predictive ability for the OS rates in the two CGGA validation cohorts (Supplementary Figure 4).

## DISCUSSION

Autophagy has been reported to play a key role in tumorigenesis, progression aggressiveness, and therapeutic resistance of multiple cancers, especially glioma [8–10]. Correcting the dysregulation of autophagy-related pathways can suppress tumor growth and improve sensitivity to various therapies. Chang et al. [22] reported that honokiol-induced autophagy can promote apoptosis and inhibit GBM cell growth. Zhang et al. [23] reported that high expression of the ATG MAPK8IP1 and low expression of SH3GLB1 can suppress the proliferation, migration and invasion of glioma cells. In addition, most studies have focused more intensely on treatments of GBM targeting autophagy in recent years. Incorporation of the autophagy modulating drug temozolomide (TMZ) with concomitant radiotherapy significantly improved patient survival by inducing autophagy [12, 13]. Therefore, ATGs are promising therapeutic targets and prognostic predictors in GBM.

By leveraging advances in large-scale genome-sequencing technologies, numerous studies have investigated large numbers of molecular biomarkers for GBM [14–16]. Previous studies have investigated multiple gene expression patterns in GBM, which can be used for risk stratification, treatment guidance, and prognosis prediction [5, 6, 14–16]. However, global expression patterns based on ATGs have not been previously constructed in GBM. In this study, we first identified 72 DE-ATGs based on the TCGA database and then confirmed 3 genes significantly correlated with prognosis in univariate and multivariate Cox regression analyses. High expression levels of ITGA3, MAP1LC3A, and NRG1 were associated with poor prognosis in GBM patients. GSEA revealed that these 3 ATGs were mainly enriched in KEGG pathways related to autophagy and cancer, providing strong evidence that dysregulation of autophagy plays a vital role in the development and progression of GBM. NRG1, one of the most active members of the epidermal growth factor (EGF)-like family, encodes a membrane glycoprotein that mediates cell-cell signaling and plays a critical role in the growth and development of multiple organ systems [24]. An et al. [25] reported that NRG1 can inhibit doxorubicin-induced autophagy via multiple signaling pathways to prevent further damage from cardiomyopathy. Previous studies have demonstrated that NRG1 plays an important role in aspects of glioma development and progression, including cell survival, migration, proliferation, and metastasis [26, 27]. Recently, Yin et al. [28] investigated whether Nrg1 can regulate apoptosis and invasion in GBM via targeting by miR-125a-3p. ITGA3 encodes a preproprotein that is proteolytically processed to generate light and heavy chains that comprise the alpha 3 subunit [29, 30]. This subunit joins with a beta 1 subunit to form an integrin that functions as a cell surface adhesion molecule and interacts with extracellular matrix proteins. The literature reports that high expression of ITGA3 can promote proliferation, progression and invasion in various tumors, such as cholangiocarcinoma, thyroid carcinoma, pancreatic adenocarcinoma and glioma [31–33]. Fiscon et al. [33] demonstrated that ITGA3 was involved in ECM-receptor interactions and focal adhesion pathways and promoted the development and differentiation of GBM cells. MAP1LC3A encodes a light chain subunit that can associate with either MAP1A or MAP1B. MAP1A and MAP1B are microtubule-associated proteins that mediate the physical interactions between microtubules and components of the cytoskeleton. The expression of MAP1LC3A was reported to be suppressed in many tumor cell lines, suggesting that it may be involved in the carcinogenesis of multiple tumors, such as gastric cancer, esophageal squamous carcinoma, osteosarcoma, and glioma [34, 35]. Giatromanolaki et al. [35] reported that extensive expression of MAP1LC3A was observed in 43% of GBM samples and that upregulation of MAP1LC3A was associated with impaired autophagic activity, which may facilitate GBM carcinogenesis. In addition, Zhang et al. [36] indicated that the products of MAP1LC3A can serve as autophagic markers and indicate autophagic activity. This group used nanoparticles loaded with curcumin to initiate autophagy, which promoted antimigratory and anti-invasive effects on GBM. In summary, NRG1, ITGA3 and MAP1LC3A may serve as tumor inducers by regulating autophagy and apoptosis in GBM. These ATGs can be used as independent prognostic biomarkers and novel targets for guiding GBM therapy.

Then, we developed and validated a novel prognostic signature based on the expression of three ATGs that, compared with clinical risk factors, improves the ability to predict the survival of patients with GBM. According to the ATG-based risk score model, patients with GBM were divided into a high-risk group and a low-risk group. Patients with high risk scores had significantly poorer OS than patients with low risk scores. Therefore, more precise individualized treatment strategies for GBM patients with high risk scores can be established. These patients should receive more aggressive treatments and closer follow-up to detect recurrence.

Nomograms have been widely used in clinical practice for their intuitive visual presentation [37]. To our knowledge, this nomogram is the first to incorporate an autophagy-related signature for predicting the survival of GBM patients that was constructed and validated in large databases with long-term follow-up. In this study, we established a nomogram incorporating the autophagy signature, age, pharmacotherapy, radiotherapy, and IDH mutation status. Calibration plots based on the TCGA and CGGA databases indicated that actual survival corresponded closely with predicted survival, suggesting that the predictive performance of the nomogram was excellent. This visual scoring system could assist both physicians and patients in performing individualized survival predictions, which would facilitate the selection of better treatment options.

The present study has some limitations. First, the clinical information downloaded from the TCGA and CGGA databases was limited and incomplete. Detailed information about neuroimaging, resection extent, radiotherapy and chemotherapy were not enrolled in the nomogram. Second, the prediction model needs further validation in multicenter, large-scale clinical trials and prospective studies.

In conclusion, by assessing the global gene expression profile, we identified a reliable autophagy-related three-gene risk score model that has significant value in predicting the prognosis of GBM patients and could suggest therapeutic targets for GBM. Then, we established a novel promising prognostic nomogram model incorporating both the autophagy signature and clinical parameters for providing individualized survival prediction and facilitating the selection of better treatment strategies. Further studies in large-scale, multicenter and prospective clinical cohorts are needed to verify the prognostic model developed in this study.

## MATERIALS AND METHODS

### Data retrieval and processing

The level 3 RNA sequencing data and clinical information of GBM patients were downloaded from The Cancer Genome Atlas (TCGA, https://portal.gdc.cancer.gov/) and the Chinese Glioma Genome Atlas (CGGA, http://www.cgga.org.cn) databases, respectively. The TCGA GBM cohort was selected as the training set and contained 155 tumor samples and 5 normal samples. The CGGA cohort was selected as the validation set; Batch-1 contained 83 GBM patients and Batch-2 contained 133 GBM patients. All patients without prognostic information were initially excluded. Because the data were obtained from TCGA and CGGA, approval for our study by the ethics committee was not necessary.

### Identification of differentially expressed autophagy-related genes (DE-ATGs) and enrichment analysis

The differentially expressed genes (DEGs) in GBM and normal samples in the TCGA cohort were screened using edgeR (https://bioconductor.org/packages/release/bioc/) in R 3.5.1 [38]. Adjusted P (adj. P) values were applied to correct the false positive results using the default Benjamini-Hochberg false discovery rate (FDR) method. Adj. P < 0.01 and |fold change (FC)| > 1 were considered the cutoff values for identifying DEGs [39]. The DEGs in the TCGA cohort are displayed in volcano plots. The 232 ATGs were obtained from the Human Autophagy Database (HADb, http://www.autophagy.lu/), which provides a complete and up-to-date list of human genes and proteins involved directly or indirectly in autophagy as described in the literature from PubMed and biological public databases [40]. The intersection of the DEGs and ATGs was considered the set of significant DE-ATGs for further analysis and was then visualized via Venn diagrams.

Then, the Database for Annotation, Visualization and Integrated Discovery (DAVID, http://david.ncifcrf.gov/) was used to perform functional annotation and pathway enrichment analyses, including Gene Ontology (GO) and Kyoto Encyclopedia of Genes and Genomes (KEGG) pathway analysis, for the significant DE-ATGs [41, 42]. DAVID is an online tool for gene functional classification, which is essential in high-throughput gene analysis for understanding the biological significance of genes [43]. A P value of < 0.05 was considered statistically significant.

### Construction and evaluation of the ATG-based prognostic risk score model

The schematic diagram for constructing the risk score model was shown in Supplementary Figure 5. Univariate Cox regression analysis was first performed on the DE-ATGs in the TCGA GBM training cohort to identify the association between the expression levels of the genes and patients' OS using the survival package (http://bioconductor.org/packages/survival/) in R 3.5.1 [44]. Then, genes with a P value of < 0.05 identified by univariate Cox regression were further screened by multivariate Cox regression. Based on the Akaike information criterion (AIC), the optimal prognosis-related genes were determined to establish a prognostic risk score model for predicting OS [45]. According to the median expression levels of the prognosis-related genes, patients were divided into high and low expression groups [46]. Then, Kaplan-Meier (K-M) survival analysis using the survival package was performed to estimate the associations between the expression levels of the prognosis-related genes and OS.

The prognostic risk score model was established with the following formula: risk score = expression level of Gene_1_ × β_1_ + expression level of Gene_2_ × β_2_ +…+ expression level of Gene_n_ × β_n_; where β is the regression coefficient calculated by the multivariate Cox regression model [46]. Subsequently, a prognostic risk score was generated for each patient. All TCGA GBM patients were divided into the high-risk (high risk score) and low-risk (low risk score) groups according to the median value of their risk score. Then, a K-M survival curve was constructed to estimate the prognosis of patients with high risk scores or low risk scores, and the survival differences between the high-risk and low-risk groups were assessed by a two-sided log-rank test. The prognostic performance was evaluated by using Harrell's concordance index (C-index) and time-dependent receiver operating characteristic (ROC) curve analysis within 0.5, 1 and 3 years to evaluate the predictive accuracy of the ATG-based prognostic model with the R packages ‘survcomp’ (http://www. bioconductor.org/packages/survcomp/) and ‘survivalROC’ (https://cran.r-project.org/web/packages/survivalROC/) [37, 47]. The values of both the C-index and area under the ROC curve (AUC) range from 0.5 to 1, with 1 indicating perfect discrimination and 0.5 indicating no discrimination. Then, the performance of the ATG-based risk score model constructed by the TCGA training set was validated in the CGGA Batch-1 and Batch-2 GBM cohorts via a similar approach.

Furthermore, to determine whether the predictive power of the ATG-based prognostic model could be independent of other clinicopathologic parameters (including age, sex, new event occurrence, pharmacotherapy, Karnofsky performance score (KPS), radiotherapy, surgery, IDH status and 1p/19q status) for patients with GBM, univariate and multivariate Cox proportional hazards regression analyses were performed in the TCGA training set and the two CGGA validation sets.

### Gene set enrichment analysis (GSEA)

Gene expression levels were set as population phenotypes, and GSEA (http://software. broadinstitute.org/gsea/index.jsp) was used to assess related pathways and molecular mechanisms in GBM patients [48]. Enriched gene sets with a nominal P value of < 0.05 and a FDR of < 0.25 were considered statistically significant.

### Construction and validation of the nomogram

Following univariate Cox regression analysis, all independent prognostic factors were screened by multivariate Cox regression analysis for the construction of a prognostic nomogram to assess the probability of 0.5-, 1-, and 3-year OS for TCGA GBM patients via the rms R package (https://cran.r-project. org/web/packages/rms/) [49]. The discrimination performance of the nomogram was quantitatively assessed by the C-index and the AUC [37]. Calibration plots were also used to graphically evaluate the discriminative ability of the nomogram [47]. Finally, the prognostic nomogram was externally validated in the CGGA Batch-1 and Batch-2 cohorts. All analyses were conducted using R version 3.5.1, and a P value of < 0.05 was considered statistically significant. Hazard ratios (HRs) and 95% confidence intervals (CIs) were reported if necessary.

## Supplementary Material

Supplementary Figures

Supplementary Tables

Supplementary Table 2

Supplementary Table 3
