# Supplementary material for: Development and validation of a nomogram with an autophagy-related gene signature for predicting survival in patients with glioblastoma
Source: Aging (Albany NY). 2019 Dec 17;11(24):12246–69. doi: 10.18632/aging.102566 (PMC6949068; doi:10.18632/aging.102566)
Supplement: Supplementary Figures [file aging-11-102566-s004..pdf]

SUPPLEMENTARY FIGURES

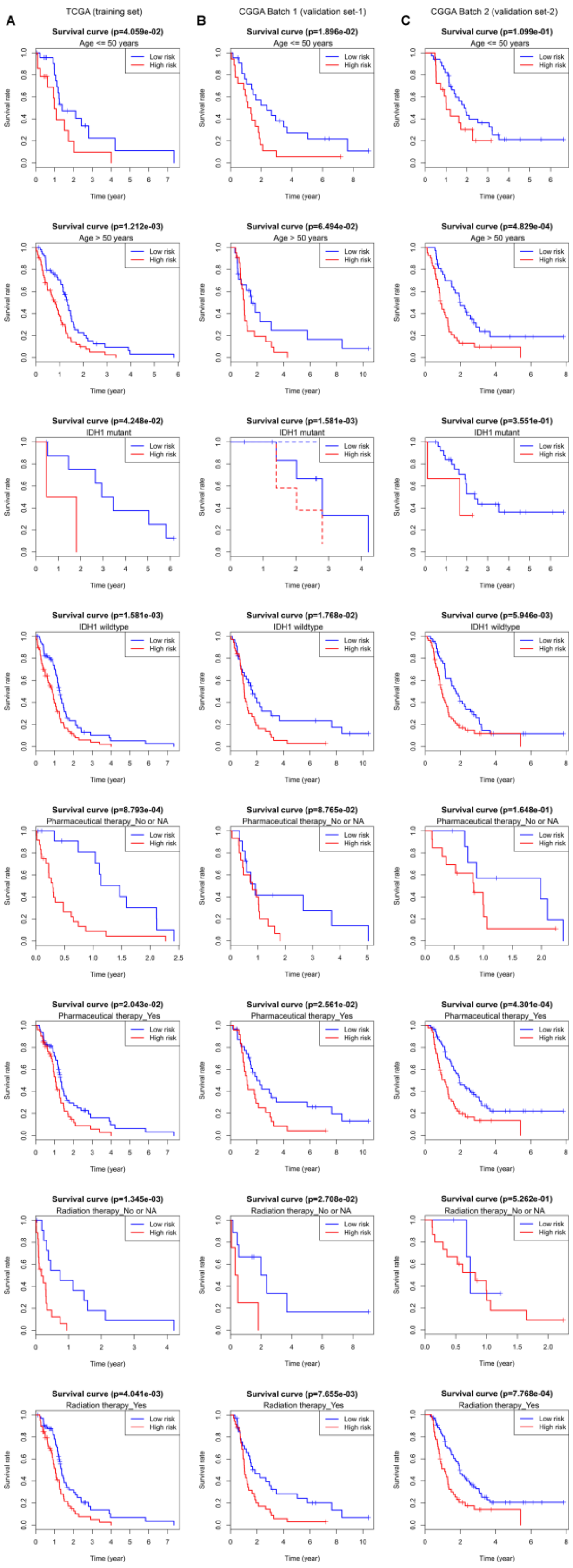

Supplementary Figure 1. Kaplan-Meier survival curves of OS according to low or high risk scores stratified by age, IDH mutation status, pharmaceutical therapy, and radiation therapy in the TCGA training cohort (**A**), CGGA Batch-1 validation cohort (**B**), and CGGA Batch-2 validation cohort (**C**).

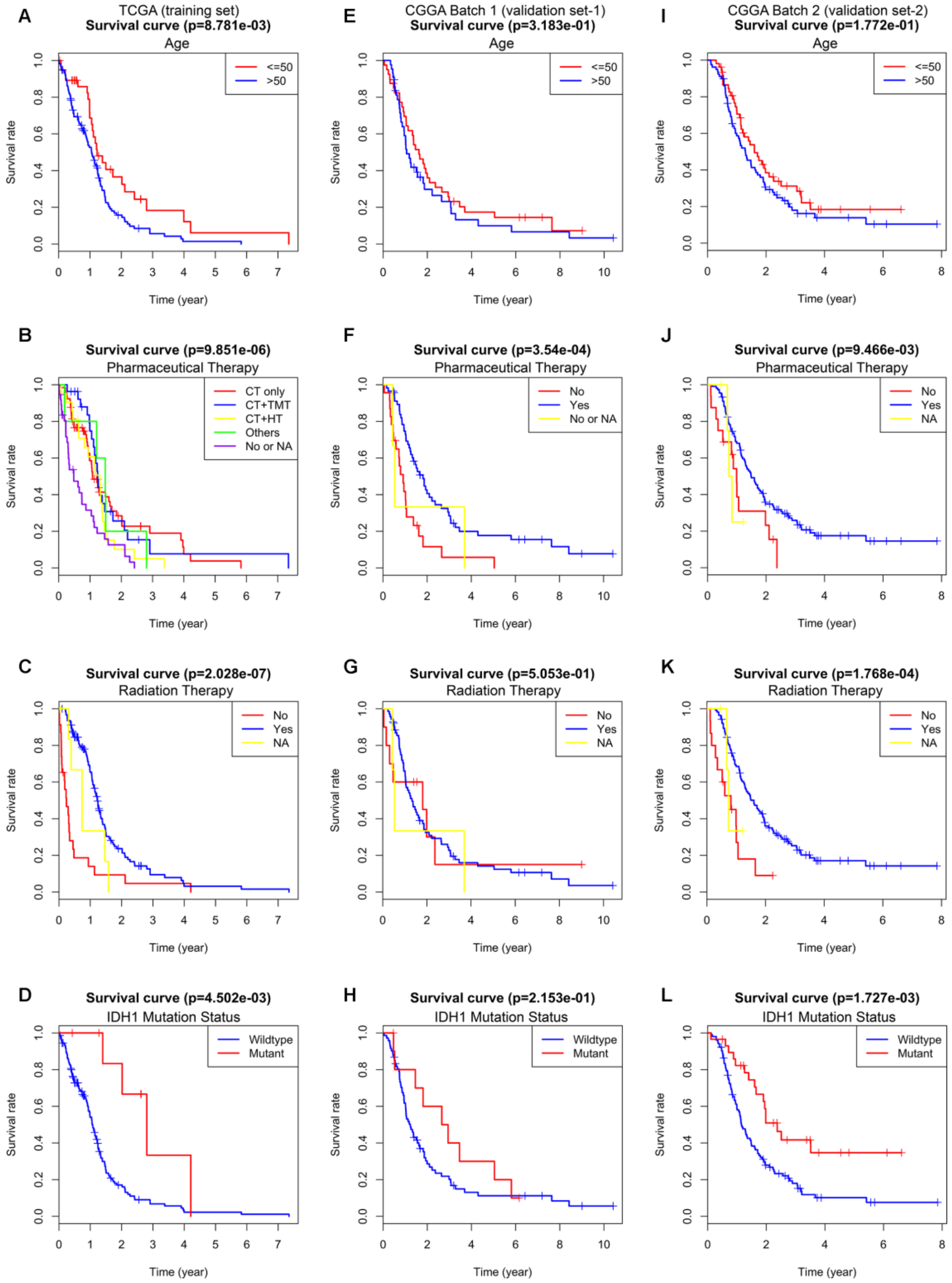

Supplementary Figure 2. Kaplan-Meier survival curves of age, pharmaceutical therapy, radiation therapy, and IDH mutation status for the OS of patients in the TCGA training cohort, CGGA Batch-1 validation cohort, and CGGA Batch-2 validation cohort.

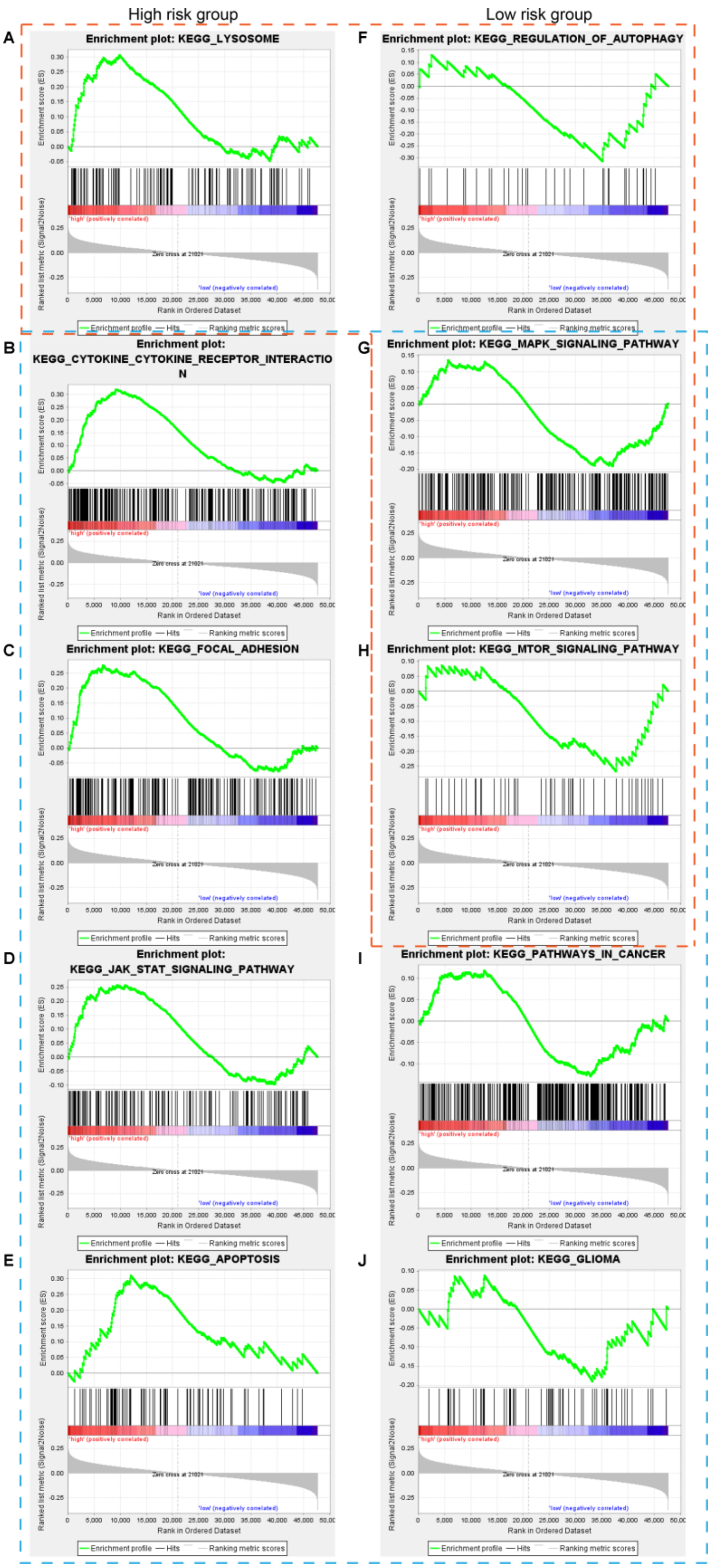

Supplementary Figure 3. Gene set enrichment analysis (GSEA) was performed between the ATG-based high-risk and low-risk groups based on the TCGA GBM cohort. Red box: regulation of autophagy and autophagy-related KEGG pathways. Blue box: pathways in cancer and their related KEGG pathways, including glioma.

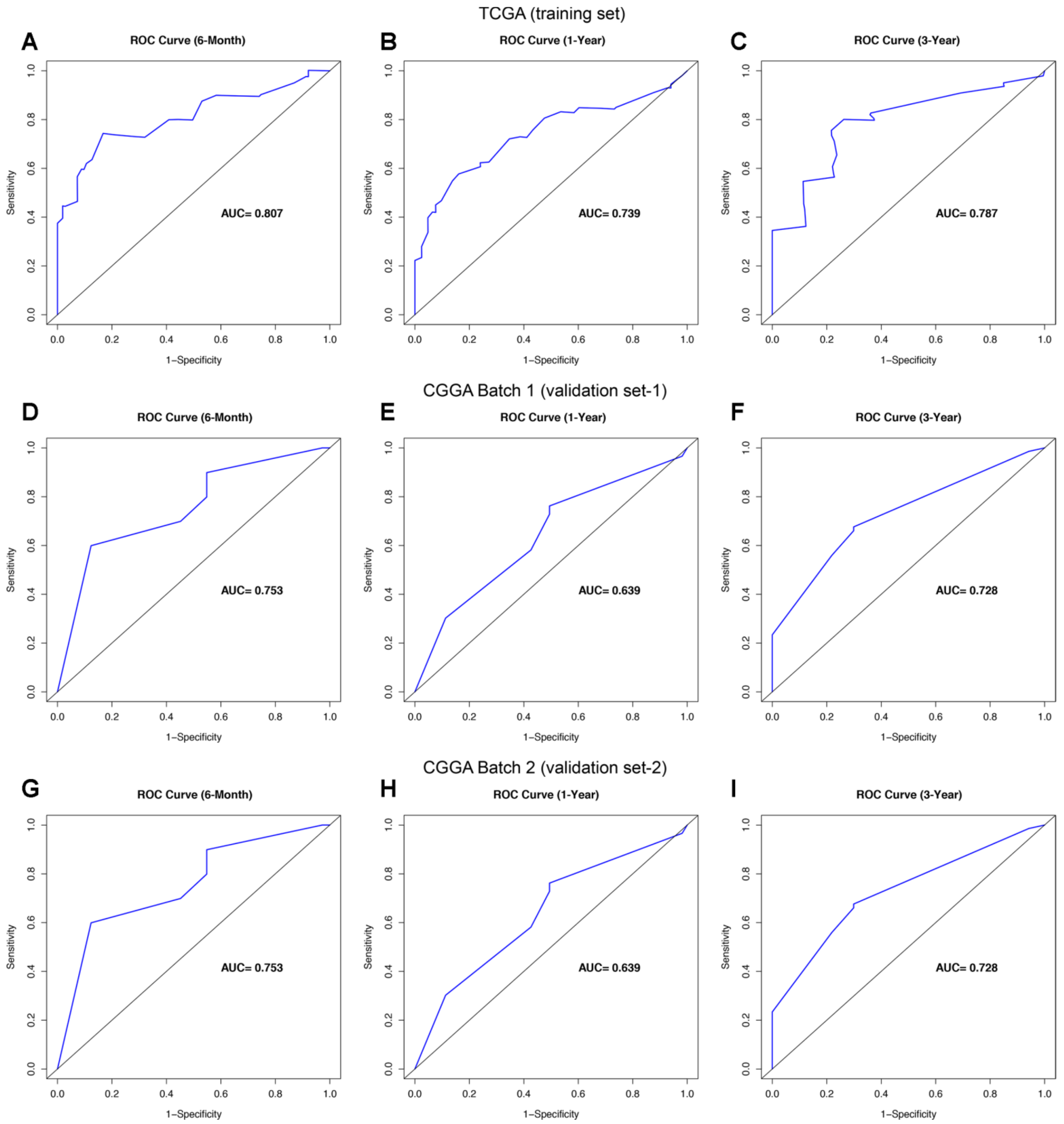

Supplementary Figure 4. The prognostic performance of the nomogram demonstrated by the ROC curve for predicting the 0.5-, 1-, and 3-year OS rate in the TCGA training cohort (**A**–**C**), CGGA Batch-1 validation cohort (**D**–**F**), and CGGA Batch-2 validation cohort (**G**–**I**).

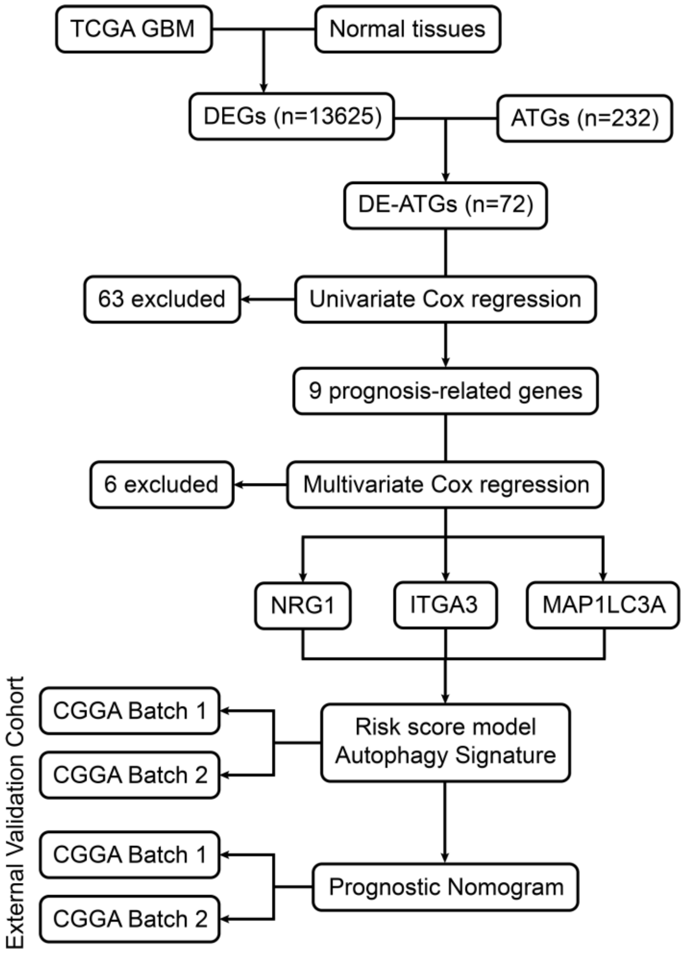

Supplementary Figure 5. The schematic diagram for constructing the prognostic prediction model based on autophagy signature.
