# Supplementary material for: Development and validation of a nomogram with an autophagy-related gene signature for predicting survival in patients with glioblastoma
Source: Aging (Albany NY). 2019 Dec 17;11(24):12246–69. doi: 10.18632/aging.102566 (PMC6949068; doi:10.18632/aging.102566)
Supplement: Supplementary Tables [file aging-11-102566-s003..pdf]

## SUPPLEMENTARY TABLES

**Supplementary Table 1. Cox regression analysis of 72 differentially expressed autophagy-related genes in gliomablastoma patients**

| Gene | Univariate analysis | | | Multivariate analysis | | |
|---|---|---|---|---|---|---|
| | HR | z | pvalue | HR | z | pvalue |
| CXCR4 | 1.12752231 | 1.36150731 | 0.17335342 | - | - | - |
| MAP1LC3C | 1.09214902 | 1.51592606 | 0.12953805 | - | - | - |
| IL24 | 1.09055155 | 1.02655736 | 0.30462894 | - | - | - |
| CASP1 | 1.09631446 | 1.05763629 | 0.29022129 | - | - | - |
| APOL1 | 1.03878724 | 0.56953867 | 0.56899063 | - | - | - |
| PARK2 | 1.1116884 | 1.05253107 | 0.29255596 | - | - | - |
| GRID1 | 0.96610039 | -0.4636662 | 0.64288692 | - | - | - |
| RB1CC1 | 1.05469152 | 0.4335599 | 0.66460806 | - | - | - |
| WDFY3 | 0.97232968 | -0.2591414 | 0.79552617 | - | - | - |
| EGFR | 0.96155822 | -0.9756129 | 0.32925635 | - | - | - |
| ERBB2 | 1.15572387 | 1.78927734 | 0.07357016 | - | - | - |
| NRG3 | 1.02157032 | 0.41012589 | 0.6817136 | - | - | - |
| HDAC1 | 0.9681987 | -0.2503851 | 0.80228957 | - | - | - |
| CASP4 | 1.27332956 | 2.31674033 | 0.02051788 | - | - | - |
| MAPK8IP1 | 1.01901845 | 0.2236201 | 0.82305292 | - | - | - |
| RPTOR | 1.06053203 | 0.55691437 | 0.57758593 | - | - | - |
| AMBRA1 | 1.10502149 | 0.88581859 | 0.37571529 | - | - | - |
| SERPINA1 | 1.15201256 | 1.96520121 | 0.04939095 | - | - | - |
| DAPK2 | 1.15644409 | 1.62702842 | 0.10373106 | - | - | - |
| CASP3 | 1.19157365 | 1.61187975 | 0.10698811 | - | - | - |
| DIRAS3 | 1.16347952 | 2.53110692 | 0.01137032 | - | - | - |
| IFNG | 1.21783781 | 1.31300654 | 0.18918075 | - | - | - |
| PINK1 | 1.11048638 | 0.9324178 | 0.35112065 | - | - | - |
| ATG2B | 1.02325887 | 0.21985061 | 0.82598751 | - | - | - |
| BIRC5 | 1.03313466 | 0.47552664 | 0.63441165 | - | - | - |
| CTSB | 1.31560381 | 2.54009925 | 0.0110821 | - | - | - |
| ITGB1 | 1.04985848 | 0.45753454 | 0.64728688 | - | - | - |
| MAPK1 | 0.92472275 | -0.7449741 | 0.45628737 | - | - | - |
| IKBKB | 1.08210872 | 0.59394047 | 0.55255193 | - | - | - |
| MAPK8 | 0.91317847 | -0.9090328 | 0.36333282 | - | - | - |
| PRKCQ | 0.93580259 | -0.8419977 | 0.39978924 | - | - | - |
| ULK1 | 1.16006405 | 1.17391162 | 0.24043043 | - | - | - |
| DRAM1 | 1.21266735 | 1.9069304 | 0.0565296 | - | - | - |
| FKBP1B | 1.37459207 | 2.92057153 | 0.0034939 | - | - | - |
| GNB2L1 | 0.83523584 | -1.2999676 | 0.19361207 | - | - | - |
| MAPK3 | 1.12040281 | 0.81243541 | 0.41654183 | - | - | - |
| NRG1 | 1.14389011 | 2.74819125 | 0.0059925 | 1.14157962 | 2.66231234 | 0.00776058 |

| | | | | | | |
|---|---|---|---|---|---|---|
| ITGA3 | 1.19112653 | 2.68707229 | 0.00720814 | 1.14941814 | 2.02128687 | 0.04325008 |
| VEGFA | 1.08260766 | 1.50294174 | 0.13285407 | - | - | - |
| MYC | 0.9135298 | -1.0194185 | 0.30800432 | - | - | - |
| ATG4A | 1.05172571 | 0.34005326 | 0.73381642 | - | - | - |
| CDKN1A | 1.10613952 | 1.36952825 | 0.17083421 | - | - | - |
| GNAI3 | 0.9559024 | -0.2984975 | 0.76532347 | - | - | - |
| FAS | 1.16693931 | 1.89196524 | 0.05849561 | - | - | - |
| PRKCD | 1.16153179 | 1.51380892 | 0.13007431 | - | - | - |
| TP73 | 0.95783626 | -0.8832974 | 0.37707559 | - | - | - |
| TMEM74 | 1.02652779 | 0.43315698 | 0.66490073 | - | - | - |
| MAPK9 | 1.11804113 | 0.81646607 | 0.41423362 | - | - | - |
| ITPR1 | 1.21698874 | 1.86942202 | 0.06156412 | - | - | - |
| CX3CL1 | 0.99173975 | -0.0990952 | 0.92106272 | - | - | - |
| IKBKE | 1.2062973 | 1.64515448 | 0.09993796 | - | - | - |
| HIF1A | 0.93383523 | -0.7361781 | 0.4616223 | - | - | - |
| TUSC1 | 1.12449246 | 1.6239062 | 0.10439583 | - | - | - |
| ULK2 | 1.0903667 | 0.79924586 | 0.42414787 | - | - | - |
| CAMKK2 | 1.10383881 | 0.71073959 | 0.47724562 | - | - | - |
| CDKN2A | 0.96348157 | -1.178491 | 0.23860092 | - | - | - |
| DAPK1 | 0.99689091 | -0.0375145 | 0.97007475 | - | - | - |
| BAK1 | 0.93815456 | -0.4304121 | 0.66689588 | - | - | - |
| NAMPT | 1.12745712 | 1.8547772 | 0.06362805 | - | - | - |
| TSC1 | 0.96762564 | -0.3495673 | 0.72666347 | - | - | - |
| EIF4EBP1 | 1.0262793 | 0.20731651 | 0.83576268 | - | - | - |
| TP53 | 0.92835436 | -0.8075141 | 0.41937034 | - | - | - |
| MAP1LC3A | 1.39894856 | 3.17692527 | 0.00148845 | 1.30823622 | 2.46357359 | 0.01375597 |
| CASP8 | 1.08199498 | 0.71583924 | 0.47409062 | - | - | - |
| RGS19 | 1.21061651 | 1.30953568 | 0.19035296 | - | - | - |
| CCR2 | 1.11426965 | 1.70918135 | 0.08741736 | - | - | - |
| PRKAR1A | 1.1494814 | 1.06072757 | 0.28881373 | - | - | - |
| GABARAPL1 | 1.13059973 | 1.18350566 | 0.23660881 | - | - | - |
| P4HB | 1.35062977 | 2.14162036 | 0.03222404 | - | - | - |
| PTK6 | 0.95966931 | -0.6245504 | 0.53226621 | - | - | - |
| BAX | 1.06447347 | 0.52869632 | 0.59701613 | - | - | - |
| TP63 | 1.12260577 | 2.22352058 | 0.02618072 | - | - | - |

Please browse Full Text version to see the data of Supplementary Tables 2 and 3.

**Supplementary Table 2. GSEA reports for high ITGA3, MAP1LC3A, and NRG1 expression groups in TCGA-GBM cohort.**

**Supplementary Table 3. GSEA reports for ATG-based high-risk and low-risk group in TCGA-GBM cohort.**
